# Are There Benefits from Teaching Yoga at Schools? A Systematic Review of Randomized Control Trials of Yoga-Based Interventions

**DOI:** 10.1155/2015/345835

**Published:** 2015-09-28

**Authors:** C. Ferreira-Vorkapic, J. M. Feitoza, M. Marchioro, J. Simões, E. Kozasa, S. Telles

**Affiliations:** ^1^Department of Physiology, Laboratory of Neurophysiology, Federal University of Sergipe (UFS), Avenida Marechal Rondon, s/n, Jardim Rosa Elze, São Cristóvão, 49100-000 Aracaju, SE, Brazil; ^2^Department of Psychology, FASE*\*UNESA, Aracaju, SE, Brazil; ^3^Trika Research Center, Loei, Thailand; ^4^Hospital Israelita Albert Einstein, São Paulo, Brazil; ^5^Department of Psychobiology, Federal University of São Paulo, São Paulo, Brazil; ^6^Indian Council of Medical Research Center for Advanced Research in Yoga and Patanjali Research Foundation, Bengaluru, India

## Abstract

*Introduction*. Yoga is a holistic system of varied mind-body practices that can be used to improve mental and physical health and it has been utilized in a variety of contexts and situations. Educators and schools are looking to include yoga as a cost-effective, evidence-based component of urgently needed wellness programs for their students. *Objectives*. The primary goal of this study was to systematically examine the available literature for yoga interventions exclusively in school settings, exploring the evidence of yoga-based interventions on academic, cognitive, and psychosocial benefits. *Methods*. An extensive search was conducted for studies published between 1980 and October 31, 2014 (PubMed, PsycInfo, Embase, ISI, and the Cochrane Library). Effect size analysis, through standardized mean difference and Hedges'g, allowed for the comparison between experimental conditions. *Results and Conclusions*. Nine randomized control trials met criteria for inclusion in this review. Effect size was found for mood indicators, tension and anxiety in the POMS scale, self-esteem, and memory when the yoga groups were compared to control. Future research requires greater standardization and suitability of yoga interventions for children.

## 1. Introduction

Yoga is an ancient mind-body practice which originated in India more than 2.000 years ago and is described systematically early on (Patanjali's Yoga* Sutras*,* circa* 900 B.C.). Although, according to traditional scriptures, its ultimate goal is to achieve a unified state of consciousness and self-realization, yoga may be used to improve overall health and well-being [1]. Yoga involves different techniques such as physical postures (*asanas*), controlled breathing (*pranayamas*), deep relaxation (*yoganidra*), and meditation [1]. These techniques seem to have specific influences on one's psychological state [2, 3] and the research on the psychophysiological benefits of yoga and meditation on adults has demonstrated improvements in emotional self-regulation with consequent reductions in depression, stress, anxiety levels [2–11] and posttraumatic disorder [12] as well as improvements in mood [13], quality of life, and well-being [14–16]. However, in spite of the positive effects of yoga on mental health, this practice is not just limited to therapeutic use and has been utilized in a variety of situations and contexts, including educational and school settings, where teaching students about wellness and health accompanies the primary aim of academic instruction [17].

According to United Nations [18], children and adolescents around the world spend an average of 10 to 15 years at school. As a result, schools hold the potential to teach about healthy habits from an early age and promote children's health and well-being. For children who have to deal with stressors, anxiety, traumas, abuse, learning disabilities, and even bullying, the discipline developed by practicing contemplative techniques could be the difference between failure and success, both in their professional and personal life. [19]. Furthermore, according to Noggle et al. [20], the age of onset of most mental health disorders in adults occurs during childhood and adolescence, with around 7.5% of adolescents meeting DSM-IV-TR criteria for one or more mental health conditions.

The solution for dealing with stressors, anxiety, and learning disabilities certainly depends on many factors; however, evidence suggests that some of these problems may be eased by mind-body practices, which have been shown to redirect attention, improve concentration, increase self-control, and provide people with reliable and healthy coping mechanisms [19]. Yet, the efficacy of such practices among children is unclear and evidence is insufficient. A review conducted by Galantino et al. [21] found that there was evidence for the benefit of yoga in the pediatric population in physical rehabilitation, but a recent meta-analysis concluded that the data on the clinical applications of yoga among the children are uncertain [22]. Authors state that while most studies were suggestive of benefits, results were based on low quality trials.

Studies utilizing yoga in school settings have been shown to benefit children and adolescents [17]. According to Khalsa et al. [23], a yoga program might help children recover their self-esteem and confidence, restore their mental health, promote positive attitudes, improve concentration, and reduce stress and anxiety. Unfortunately, traditional curricula focus primarily on intellectual development, and schools have progressively been losing the capacity to adopt health-focused programs. The ability to cope with stress and anxiety (due to psychosocial demands) and to maintain physical and mental health is priceless in any spheres of an individual's life, including education. Students must be healthy in order to learn, and academic accomplishment has been shown to be related to health status. Consequently, there is urgent need to develop and investigate cost-effective and evidence-based wellness programs that can be delivered in school settings.

Therefore, the primary goal of this study was to systematically examine the available literature on yoga interventions exclusively in school settings. The objective of this report was to review methodological quality among selected studies, exploring the evidence of yoga-based interventions regarding academic, cognitive, and psychosocial benefits, and to contribute to the study of low-cost, health-focused alternative programs for children and adolescents in school settings.

## 2. Methods

### 2.1. Searching

Studies were identified by searching PubMed, PsycInfo, Embase, ISI, and the Cochrane Library. A wide search was conducted for studies published between 1980 and October 31, 2014, using the following terms or key words:* yoga*,* school*,* education*,* and children* alone and in combination with additional terms such as* program*,* intervention*, and* yoga-based*. A manual evaluation of reference lists of relevant studies and reviews was also conducted. All articles related to the subject* yoga at schools* were selected for additional examination.

### 2.2. Selection

Peer-reviewed, published manuscripts were considered. Studies were selected if (1) they included a yoga or yoga-based intervention, (2) the intervention was restricted to school settings (integrated into the school schedule or after class), (3) they included children and adolescents (ages 5–18), (4) they included an evaluation of anxiety, depression, stress, or other psychological measures such as mood indicators, self-esteem, confidence, and quality of life at both preintervention and postintervention, (5) they included the assessment of academic or cognitive performance as a consequence of the yoga intervention (pre- and postintervention), (6) the research designs were pilot studies, quasi-experimental designs, or randomized designs and included control groups with no interventions or an active control (comparative intervention), and (7) they were written in English. Exclusion criteria comprised (1) studies that utilized only meditation or relaxation techniques without the physical components such as postures (*asana*) or controlled breathing (*pranayama*) (MBSR based programs usually have yoga as part of the practice but only MBSR studies in which yoga is highlighted or is the main component of the program were included in this review whose intention is to cover primarily yoga), (2) yoga-based programs for children with learning disabilities or any diagnosed mental disorder, and (3) dissertations and conference presentations.

Authors screened abstracts to identify articles that meet inclusion criteria. Potential articles were then evaluated for inclusion. To conduct the study, all data was collected and evaluated in terms of selection criteria, procedure, participants, intervention, methodology, assessment tools, and outcomes. Follow-up and results were also assessed. To evaluate or discuss non-RCTs was not part of the review's scope. Other reviews have mentioned non-RCTs, including methodology and possible bias [17]. The review has been prepared using preferred reporting criteria for systematic review guidelines (PRISMA) [24].

The quality and reliability of the randomized control trials (RCTs) were evaluated according to the evidence levels recommended by the Oxford Center for Evidence-Based Medicine [25].  Table 1 shows the classification of scientific evidence in systematic literature reviews. Four authors conducted the literature searching (C. Ferreira-Vorkapic, M. Marchioro, and S. Telles) and study selection (C. Ferreira-Vorkapic, M. Marchioro, and E. Kozasa).

After the first appraisal, another examiner (J. Simões) evaluated the selected RCTs and* kappa* coefficient was calculated in order to estimate reliability of data collection methods. The observed kappa score of *r* = 0.9 suggests great consistency of agreement between investigators.

For the effect size calculation, the means and standard deviations (postintervention) from experimental and control groups were obtained directly from the studies. The comparison between experimental conditions was carried out after the analysis of the pooled effect size by the generic inverse variance method (random effect model) through standardized mean difference and Hedges' g. Hedges' g effect is the difference between the two means (postintervention for the experimental and the control groups) divided by the pooled standard deviation. Ninety-five percent confidence intervals were computed for all variables.

Two studies [19, 26] were excluded from the effect size analysis due to insufficient data (mean values not provided).

## 3. Results

### 3.1. Description of Studies

**Figure 1:**
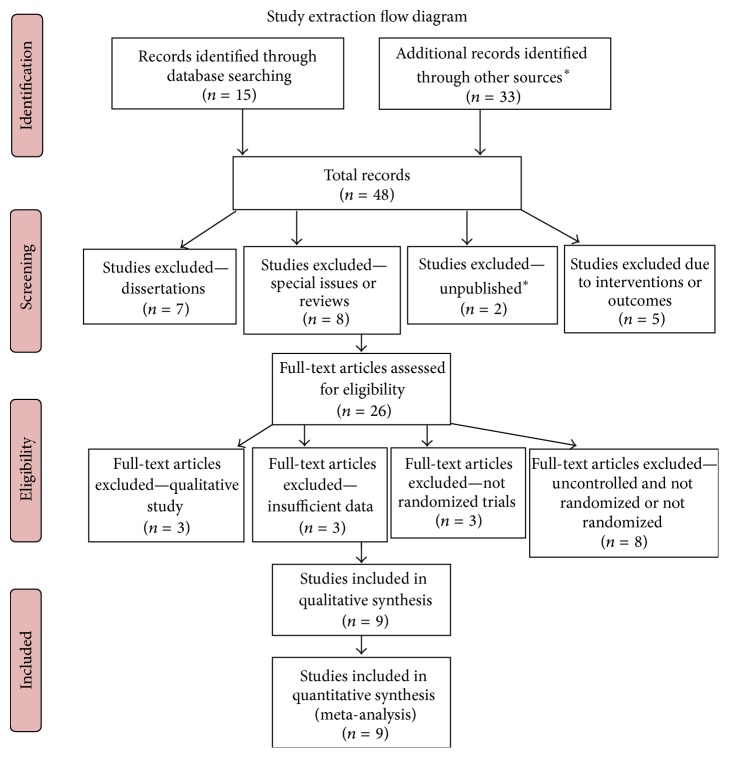
Criteria for inclusion in the review. ^*∗*^Articles sent to the authors by other researchers.

Of the 48 studies identified, 9 randomized control trials met criteria for inclusion in this review, as illustrated by Figure 1 (PRISMA Guidelines Study Extraction) [24].  Table 2 shows the PRISMA 2009 checklist. Selected randomized control trials (RCTs) are displayed in Table 3.

Generally, the RCTs had low AHRQ evidence scores, with most studies receiving a score of 2b due to the lack of blindness and follow-up, reflecting the reduced quality of reporting in these studies.

All results and conditions of the studies are summarized in Table 3.

Due to the limited number of RCTs and the great heterogeneity of the variables (ununiform constructs), the analysis of the effect size of specific measures could only be performed on studies that observed the same variables such as mood, tension, anxiety [20, 23], self-esteem [23, 27], and memory [28, 29] when comparing the yoga to control groups.

**Figure 2:**
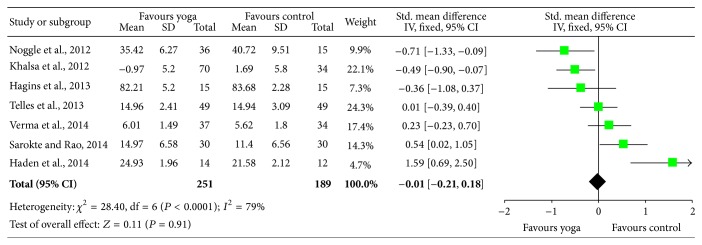
Plot of the general effect size in the selected studies.

After an overall effect size calculation of each study (except for [19, 26]), the effect sizes obtained from similar measures were grouped together: mood, tension, anxiety, self-esteem, and memory.  Figure 2 shows the forest plot of the general effect size in the selected studies. The general plot shows divided results with half of the studies favoring yoga and the other half favoring control. *P* value for overall effect is not significant (*P* = 0.91), but this is probably due to heterogeneity of the variables.

**Figure 3:**
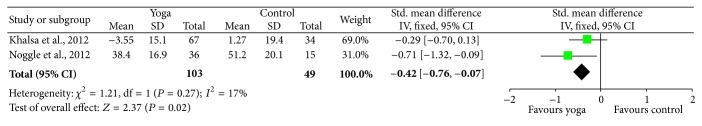
Profile of Mood States (POMS) general score effect size.

**Figure 4:**
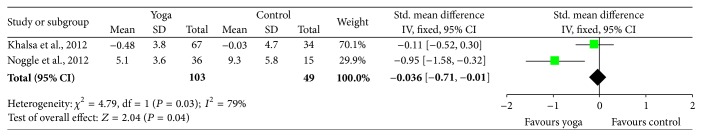
Profile of Mood States (POMS): subitems tension and anxiety effect size.

Effect size from mood state indicators (POMS) was calculated from Khalsa et al., 2012 [23], and Noggle et al., 2012 [20]. Results indicate that the yoga group showed significantly better scores in the postintervention condition (*P* = 0.02) (Figure 3). The same scale shows a second significant effect for the subitems tension and anxiety also after the yoga practice (*P* = 0.04) (Figure 4).

**Figure 5:**
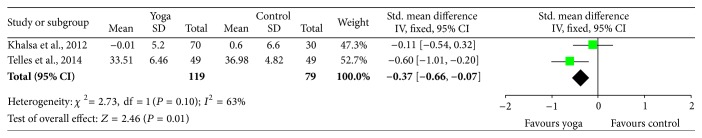
Self-esteem effect size.

A third comparison was carried out for the variable self-esteem [23, 27]. Results show greater self-esteem perception in the postintervention condition for the yoga group (*P* = 0.04) (Figure 5).

**Figure 6:**
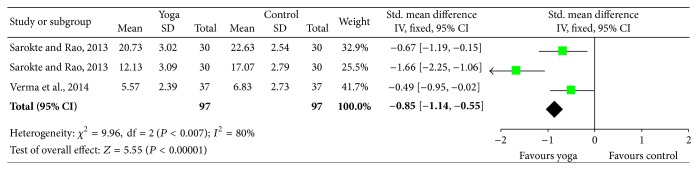
Memory effect size.

Effect size for memory was also analyzed in [28] and Verma et al., 2014 [29]. Both studies utilized memory assessment tools, but Sarokte and Rao [28] used two different instruments. The results show increased memory performance for the yoga group (*P* < 0.00001) (Figure 6).

Since the number of selected RCTs is reduced and other interesting results were observed during the review process (that could not provide for an effect size calculation), such as negative effects or different variables, the studies are individually described in the following sections in order to provide details, similarities, and differences between them.

### 3.2. Outcome Analysis

#### 3.2.1. Yoga and Psychological Well-Being

Six RCT studies observed the effects of a yoga program on psychological well-being in school settings.

In [30], authors sought to determine the effects of yoga on children's emotional and behavioral variables using a comparison group consisting of physical education (PE) classes. Thirty middle school children were randomized to participate in either a school-based* ashtanga* yoga program or PE class three times a week for 12 weeks. Yoga classes consisted of opening rituals (3–7 min), asana practice (30 min), seated meditation (2–5 min), and guided relaxation (4 min).

Emotional and behavioral functioning was measured before and after intervention utilizing the following tools: Positive and Negative Affect Scales (PANAS), the Child Behavior Checklist (CBCL), the Revised Parent Rating Scale for Reactive and Proactive Aggression (R-PRA), and the Self-Perception Profile for Children (SPPC). Results show no significant changes between groups in self-reported positive affect, global self-worth, aggression indices, or parents' reports of their children's externalizing and internalizing problems. However, negative affect increased for those children participating in yoga when compared to the PE program. Authors offer a few explanations for these findings: (1) the first contact with yoga may be demanding for children at this age (sixth grade) and as a result may increase stress levels in the short term; (2) the “dose” and type of yoga may not have been satisfactory; (3) one of the outcomes of yoga practice may be greater self-awareness and mindfulness and these variables were not assessed in the current study; and (4) the sample size was small.

Khalsa et al., 2012 [23], evaluated mental health benefits of yoga for adolescents in secondary school. Students were randomly assigned either to regular physical education classes (PE) or to 11 weeks of yoga practice based on the Yoga Ed program. Typical 30-minute Yoga Ed sessions included 5-minute initial relaxation and breathing techniques, 5-minute warm-up, 15 min of yoga poses, and 5-minute closing relaxation. Each session also had a theme that was discussed by the instructor, such as yoga philosophy and methodology, nonviolence, mind-body interactions and awareness, body systems, stress management, emotional intelligence, and similar topics. Psychological well-being was assessed using the Self-Report of Personality (SRP) version of the Behavior Assessment Survey for Children Version 2 (BASC-2), the Profile of Mood States-Short Form (POMS-SF), the Resilience Scale (RS), the Perceived Stress Scale (PSS), and the Inventory of Positive Psychological Attitudes-32R (IPPA).

Outcome measures revealed that yoga participants showed statistically significant differences over time relative to controls on measures of anger control and fatigue/inertia. Most outcome measures exhibited a pattern of worsening in the control group over time, whereas changes in the yoga group over time either were minimal or showed slight improvements. Authors explain that while statistically significant differences between groups were found for only a few outcome measures, each of these favored the yoga group. On most measures, findings suggested relatively small positive effects in the yoga group but marked declines in the control group. A few study limitations such as inadequate psychometric tools and short duration of the program might be responsible for the observed outcomes.

Noggle et al. [20] observed the effects of a yoga program on psychological well-being, psychological attitudes, and self-regulatory skills on 51 high school students. The overall scope of this study was to evaluate the psychological benefits of a yoga program conducted within the school curriculum for adolescents and compare them to the regular physical education classes (PE). This differed from Khalsa et al., 2012 [23], as Noggle et al. [20] focused on scales developed for normative adolescent populations and based their intervention on a different yoga style (Kripalu Yoga). Students participated in both interventions two to three times a week for 30 minutes. Yoga practice consisted of 5-minute centering and breathing exercises, 5-minute warm-up, 15 minutes of yoga postures/exercises, and 5-minute closing relaxation. The authors stated that the yoga intervention developed for this study might become a standardized Kripalu-based program for use in research within the school setting. After 10 weeks of program, students attending physical education classes showed decreases in primary outcomes (Profile of Mood States-Short Form and Positive and Negative Affect Schedule for Children). Yoga students were maintained or improved. Total mood disturbance improved in yoga students and worsened in controls as did Profile of Mood States-Short Form (POMS-SF) Tension-Anxiety subscale. Although positive affect remained unchanged in both, negative affect significantly worsened in controls (PE), while it improved in yoga students. Secondary outcomes, such as the Resilience Scale, the State Trait Anger Expression Inventory-2, and the Child Acceptance Mindfulness Measure were not significant. While the study has specific limitations regarding the real-world applicability of an education setting (sample size, group-randomized trial, observer bias, and minor irregularities), results suggest preventive benefits in psychosocial well-being from the Kripalu yoga program at school.

Ramadoss and Bose, 2010 [19], observed the effects of a yoga-based transformative life skills program (TLS) on stress levels and self-control of 387 high school students. The control group did not receive the TLS protocol, while among the intervention groups students received one, two, or five classes per week. Each yoga session lasted 15 minutes and included an orienting opening bell, focused breathing (*pranayama*), silent sitting meditation, sun salutations, posture (*asana*), rhythmic breathing, silent sitting, and a closing bell. Outcome measures were stress and self-control, evaluated by the Perceived Stress Scale and Tangney's Self-Control Scale. Results show that the intervention group demonstrated a slight decrease in stress and maintenance in self-control. In contrast, in the control group, there was no significant change in stress and a nonsignificant trend toward deterioration of self-control. The study indicates an improvement in stress, self-control, and self-awareness after 18 weeks of a yoga program, compared to a control group. Although the study has specific strengths such as qualitative feedback from students and teachers, an appropriate sample, and a condensed and effective yoga practice (which might have been the reason for the positive outcomes), however, blinding protocol was not clear and authors could have explored other psychological variables, such as anxiety and depression, since the study was conducted with vulnerable teens.

White [26] investigated the efficacy of eight-week mindfulness training through yoga with 155 middle school girls (mean age of 9.9 years) on stress and coping abilities, self-esteem, and self-regulation. A randomized group design randomly assigned two public schools to either the intervention or wait-list control group. The experimental group met one day per week, for one hour, and completed 10 minutes of yoga homework six days a week. The Mindful Awareness for Girls through Yoga program, utilized in this study, was adapted from Kabat-Zinn's Mindfulness Based Stress Reduction (MBSR) [31] and focused on the yoga portion. The 10-minute practice comprised ringing* tingsha* bells, breathing\sitting meditation, yoga warm-up, postures, and comments. Psychometric tools consisted of the Feel Bad Scale, the Schoolagers' Coping Strategies Inventory, the Global Self-Worth Subscale of the Self-Perception Profile for Children, and the Healthy Self-Regulation Subscale of the Mindful Thinking and Action Scale for Adolescents.

Authors reported no significant differences between groups. In addition, over time, the intervention group was more likely than the control group to report higher perceived stress scores (although no increase in the frequency of stressors was found) and greater frequency of coping with stress. As the authors state, as self-awareness progresses with yoga practice, children may become aware of difficult emotions. This may lead to increased perceived stress at first but subsequently may result in better means to cope with such feelings. Both groups reported significantly greater self-esteem and self-regulation. According to the authors, the negative outcomes regarding perceived stress might be due to (1) use of inadequate psychometric tools, (2) the fact that awareness of stress might have facilitated coping and that this increased awareness of stress might also have precipitated more stress, and (3) the fact that increase in stress might be transient and part of the process of becoming mindful. Study limitations include a homogeneous sample (only girls), the quality of intervention (due to a large sample size), and, especially, the fact that a greater part of the practice was done as homework and children were unattended during most of the time.

Lastly, in a 15-week study, Hagins et al. [32] examined the effects of yoga compared to a physical education class (PE) on physiological responses [based on the blood pressure (BP) and heart rate (HR)] to behavioral stressor tasks such as Mental Arithmetic Task (MAT) and Mirror Tracing Task (MTT) on 30 middle school students. After initial screening, students were randomly assigned to either PE classes or yoga course. During 15 weeks, students participated in yoga program or PE classes. Both interventions occurred three times a week and each session lasted approximately 50 minutes. The yoga class consisted of an opening ritual (centering and conscious breathing), asana practice (posture), seated meditation, and guided relaxation.

The authors stated that the two behavioral stressor tasks (Mental Arithmetic Task and Mirror Tracing Task) utilized in this investigation had been previously used successfully in studies of stress reactivity in children. The MAT consisted of simple arithmetic counts during a specific period of time. In the MTT, participants had to trace a star using only a mirror version of the star for guidance. Participants had to trace the star as many times as possible without any errors during three minutes. Systolic and diastolic BP and HR were obtained during the tests through an automated blood pressure cuff. The procedures and measures in pre- and posttests were the same. Pretesting occurred one to two weeks before the start of the intervention and posttesting occurred one to two weeks after the final class. After data analysis, authors concluded that the yoga program did not reduce stress reactivity compared to a physical education class. Furthermore, statistical analysis comparing the first stressors (MAT versus MTT) used during the pretest found that there were no significant differences in BP or HR values relative to the type of stressor used. Besides, the difficulty in finding differences in BP or HR in the participants might be due to their good health, especially after such a short time intervention. The authors concluded that the results do not support the idea that benefits from yoga are derived from a mechanism related to increased regulation of the autonomic nervous system. As in other studies, the results observed here may be related to the way yoga practice was applied and a failure to directly address the issue of reaction to perceived stress.

#### 3.2.2. Yoga and Cognitive Function

Three RCT studies observed the effects of a yoga program on different cognitive functions, such as attention, memory, and developmental abilities, in school settings.

A three-month study by Sarokte and Rao [28] observed the effects of a yoga program and Ayurvedic medicine (*Medhya Rasayana*) on mental state and cognitive functions of 90 students between the ages of 10 and 16.* Medhya Rasayana* (which means “a preparation which prevents mental and intellectual disorders” in Sanskrit) was administered every day in the morning and evening and students in the yoga group were asked to perform yogic practices regularly in a specific order. The practice included postures (asanas), breathing techniques, and meditation. The frequency or duration of each individual practice is not mentioned. In the control group, no intervention or treatment was given. The group treated with* Medhya Rasayana* showed significant and most significant changes in the objective variables measured (short-term memory test pictures and serial recall effects test). The yoga group showed significant changes with respect to subjective and objective parameters in mini mental status scale. The first follow-up also shows a greater positive impact in the Ayurveda group when compared to the yoga group. The authors concluded that* Medhya Rasayana* brings faster improvements in memory when compared to yoga. However, the yoga group was asked to perform yogic practices but it is not certain if these children were really practicing yoga at home, and if so, the duration and frequency are unclear. Although the administration of the Ayurvedic drug was also not controlled (*Medhya Rasayana* was given twice a day, possibly by the parents), it is much easier to have 12-year-old boys taking medicine than performing yogic practices, which takes discipline, effort, and time. Yet, the study shows interesting results, demonstrating that both interventions might be useful to improve cognitive functions.

Telles et al. [27] observed the effects of yoga or physical exercise on physical fitness, cognitive performance, self-esteem, and teacher-rated behavior and performance in 98 school children between the ages of 8 and 13. After initial randomization and group allocation, participants were assessed for physical fitness (Eurofit physical fitness test), performance in the Stroop task (the Stroop color-word naming task), self-esteem, and analog scales (attention, punctuality, behavior with friends, and behavior with teachers) rated by the teachers. Both groups were assessed at the end of twelve weeks. Yoga practice involved* pranayamas* (yoga breathing techniques),* sithilikarana vyayama* (loosening exercises),* asanas* (postures), chanting, and yoga relaxation techniques and lasted three months with a frequency of five times a week (45 minutes per day, during school hours). Physical exercise had the same time and frequency and consisted of jogging in place as well as bending and spinal twists. In this study, authors placed an emphasis on the differences between yoga and physical exercise, with this difference being the importance of awareness, relaxation, and breathing regulation in yoga.

After testing for the differences between groups, social self-esteem was the only variable with significant changes, increasing in the physical exercise group. pre and post values within each group also showed significant changes in cognitive function. In the Stroop task, both groups showed an increase in word scores, color scores, and color-word scores. The physical exercise group showed reduced interference raw scores and an increase in interference scores. In addition, both groups showed an improvement in obedience, academic performance, attention, punctuality, behavior with friends, and behavior with teachers.

The authors suggest that the improved scores might be due to better aerobic fitness (observed in both groups). Also, the increase in interference T scores in the physical exercise group suggests reduced flexibility and ability to respond to the task demands after this intervention. Physical activity and yoga also separately improved emotional well-being in both groups, but the underlying mechanisms are not clear. The study has limitations such as the fact that the yoga and the physical exercise programs had to fit in the school schedule, which could have produced differences in outcomes. There was also no follow-up.

Finally, Verma et al., 2014 [29] observed significant improvements in measures of mental ability and memory in an experimental yoga group of high school students, from 11 to 15 years of age, randomly divided into a yoga and a control group (*n* = 82). All children were tested before and after the 12-week intervention (or control), using the test battery of Cognition Function tests (CFTs), an Indian adaptation based on Guilford's Structure of Intellect Model. Yoga sessions were conducted for 45 min, five days a week within the school setting. The practices included chanting,* asanas* (postures), and* pranayamas* (breathing techniques). Significant improvement was observed in measures of mental ability and memory only in the experimental group, specifically cognitive processes such as attention, perception, and observation. The authors suggest that the observed results (on memory scores) indicate that yoga affected only primary processing of visual inputs. Yoga intervention was well accepted, particularly due to its short duration, making it easy to be successfully incorporated into the school curriculum.

## 4. Discussion

This review systematically examined the literature on yoga in school settings, exploring the evidence of yoga-based interventions on different psychological variables and cognitive functions.

Forty-eight peer-reviewed, published studies in which yoga was taught to school-aged children in a school setting were identified. Inclusion criteria included only randomized control studies (i.e., the control group had no intervention or an active control) in which yoga (and not just meditation) was taught and the effects on psychological well-being or cognitive functions were analyzed. After wide qualitative and quantitative synthesis, nine studies were selected.

Regarding the effects of yoga on psychological well-being, of the six studies, three of them support the benefits of yoga or yoga-based programs for children in school settings. Khalsa et al. [23] observed that yoga participants showed statistically significant differences over time relative to controls on measures of anger control and fatigue/inertia. Noggle et al. [20] also observed preventive benefits in psychosocial well-being (anxiety and negative affect) for students enrolled in a yoga program. In Ramadoss and Bose [19], only the yoga group demonstrated a slight decrease in stress while maintaining self-control.

In contrast, Haden et al., 2014 [30] and White [26] observed a significant increase in perceived stress in the yoga group compared to the physical education and control groups, respectively. However, in White [26], both groups (yoga and control) reported significantly greater self-esteem and self-regulation over time. Lastly, Hagins et al. [32] found that yoga did not reduce stress reactivity, compared to a physical education class, when students were submitted to stressor tasks.

The analysis of the effect size for psychological well-being showed that an effect size was found for mood state indicators (POMS), demonstrating that the yoga group showed significant better scores in the postintervention condition. The same scale showed a second significant effect for the subitems tension and anxiety. The variable self-esteem also showed effect size and better results in the postintervention condition for the yoga group.

The evidence for the benefits of yoga in adults, whether healthy or suffering from mental disorders, is significant [4, 16]. However, due to the reduced number of randomized trials in school settings and the conflicting findings, no definitive conclusions can be drawn from these studies with children but rather indications and suggestions based on significant and reliable but isolated results. The following subsections discuss some of the positive and negative results.

### 4.1. Initial Negative Effect and Insignificant Results

The negative effects of yoga observed in some of the studies here might be explained to some extent by (1) the adaptation process, (2) attentional control, and (3) inadequacy of yoga practice for children.

#### 4.1.1. Becoming Mindful

The practice of yoga requires effort and discipline. A child's first contact with yoga is often demanding. When yoga is added to a child's already existing academic and extracurricular activities, the child may experience higher levels of stress in the short term. According to Hayes and Feldman [33], this temporary increase in stress may also be part of the process of becoming mindful as individuals begin to recognize the typical habits of the reaction to stress. In order to confirm if the initial increase in stress is part of an adaptation process, the persistence of stress levels should be examined later on. Additionally, accomplishment in yoga depends on acquired self-confidence. Benavides and Caballero [34] have demonstrated that participants in yoga show increased self-worth but demonstrated that this is dependent on one's confidence. At first, initially, trying something new at which a person is not skilled may increase feelings of inadequacy. According to Kaley-Isley et al. [35], individuals progressing through the stages of change from precontemplative to contemplative and active often experience more distress in the transitive contemplative stage where there is awareness of the need to change, but the person has not yet developed the means or the mastery to do so. Some authors suggest that this finding would reverse with a longer dose of the intervention [35]. Kaley-Isley et al. [35] concluded that although there are a few case reports of adverse events related to the use of yoga in adults [22], there is a need to conduct controlled studies in which systematic data could be gathered regarding any adverse effects of yoga with adults, children, and adolescents.

#### 4.1.2. Poor Attentional Control

Yoga techniques such as breathing and meditation require attentional control, an executive function that is still not mature in children and adolescents. As the frontal lobes mature [36], children's capacity to exercise attentional control increases [37], but the ability remains much poorer in children than in adults [38]. Paradoxically, yoga has been found to improve attention in adults and children [8, 39–42]. Therefore, yoga practice should be specifically adapted to children so they can really benefit from the positive effects observed in adults given their brain maturity.

#### 4.1.3. Inadequacy of Practice and Methodology

The duration of yoga practice observed in some of the studies might not be suitable for children due to their inability to control attention and reduced discipline. In studies where the yoga practice has been found beneficial for the students [19, 20, 23], sessions were short and condensed, lasting from 15 to 30 minutes. Additionally, many of these studies compared the effects of yoga to exercise (physical education) and have observed very similar results. A good methodological approach would consider different intervention groups and a control group.

One of the outcomes of yoga practice may be greater self-awareness and mindfulness, a primary difference between yoga and standard physical education, and these variables were not assessed in any of the studies. Actually, physical education and yoga can be considered as complementary and it is therefore inappropriate to try and compare one as better than the other.

### 4.2. Yoga at Schools: Why It Might Work

Although the number of RCT studies observing the effects of yoga on psychological and cognitive functions in school settings is very limited, the results seem promising. Effect size was found for mood indicators, tension and anxiety in the POMS scale, self-esteem, and memory.

This review identified three RCT studies that observed the effects of yoga-based interventions on different cognitive functions, such as attention, memory, and developmental abilities. Overall, participation in a yoga program was associated with improvements in subjective and objective parameters in mini mental status scale [28], mental ability, and memory [29] while performing the Stroop task [27].

Lowered mood is associated with declines in cognitive function and, at least in adults, yoga has been reported to produce improvements in mood [43, 44]. Additionally, the practice of yoga emphasizes body awareness and involves focusing one's attention. Indeed, it has been demonstrated that yoga improves general attentional abilities in adults [8, 39–42]. Attentional focus is a key aspect of yoga. It produces similar effects as relaxation in that it tends to promote self-control, concentration, self-efficacy, and body awareness [45]. Studies with children suffering from ADHD have demonstrated significant improvements pre- to posttest in different attention scales and tasks [39, 42]. Neuroimage studies in adults demonstrate that the effect of meditation (one of yoga techniques) on gray matter was most significant in the putamen [46] and anterior cingulate cortex [47], structures involved in attention processing.

In addition, yoga practice has been shown to reduce anxiety based on reductions in psychological arousal [48] (though this variable was not measured), and studies with adults have verified that anxiety affects performance on tasks requiring attention [49]. Sarang and Telles [50] speculated that anxiety reduction was the likely basis for better performance in their study.

Two of the studies reviewed here showed significant improvements in memory tasks after a few weeks of yoga-based interventions [28, 29]. Memory improvement following the practice of breathing exercises (pranayama) [51, 52] or contemplative techniques such as meditation [53–55], a pivotal part of the yoga practice, has been widely demonstrated. Activation in the hippocampus, a subcortical structure known to be critically involved in memory processes [56], has been reported during meditative states [57, 58]. A recent study contrasting structural MRI scans of novice meditators before and after eight weeks of a meditation training program confirmed actual meditation-induced changes in regions of the left hippocampus [59]. Hippocampal differences exist between meditators and nonmeditators or actual changes of the hippocampus occur due to meditation, as revealed in structural imaging studies. Studies using positron emission tomography or functional MRI (fMRI) indicated increased brain activation (compared to baseline) during meditation in left and right hippocampal and parahippocampal regions [57, 58, 60, 61]. Even though these are studies conducted in adults, improvements in attentional networks and higher hippocampal activation might also explain the effects of yoga on cognitive functions in children.

## 5. Conclusions

This review analyzed nine peer-reviewed RCT studies, in which yoga was taught to children in a school setting. Outcome measures included psychological well-being and cognitive functions, such as attention and memory. While supportive in some studies and different variables, the utility of yoga in educational settings is uncertain due to the small number of randomized control trials in the literature. Even though only RCTs were reviewed, methodological and statistical problems might have contributed to the uncertainty: inadequate sample sizes, absence of control groups, variability in the type of yoga being taught, long duration of yoga sessions, inappropriate psychometric tools for children, and failure to measure intervening variables such as mindfulness and body awareness, which are important parts of yoga practice. This review suggests important effects of yoga-based interventions at school on both psychological status and cognitive function in some studies, but future research requires greater standardization and must deal with the problem of appropriateness; what type of yoga-based intervention is most suitable for children, specifically in terms of the frequency and duration?

## Figures and Tables

**Table 1:** Classification of scientific evidence in systematic literature reviews according to the evidence levels recommended by the Oxford Center for Evidence-Based Medicine.

Level of evidence	Grading criteria
1a	Systematic reviews of RCTs including meta-analysis.

1b	Individual RCT with narrow confidence interval.

1c	Case of series “all or nothing.”

2a	Systematic review of cohort studies.

2b	Individual cohort study and low quality RCT.

2c	Outcome research study.

3a	Systematic review of case control studies.

3b	Individual case control study.

4	Case series, poor quality cohort, and case control studies.

5	Expert's opinion.

Adapted from levels of evidence of the Oxford Center for Evidence-Based Medicine [62].

**Table 2:** PRISMA 2009 checklist.

Section/topic	#	Checklist item	Reported on page #
*Title *			
Title	1	Are There Benefits from Teaching Yoga at Schools? A Systematic Review of Randomized Control Trials of Yoga-Based Interventions.	1

*Abstract*			
Structured summary	2	*Background*. Yoga is a holistic system of varied mind-body practices that can be used to improve mental and physical health. Due to the well-known restorative effects of yoga on mental health, it has been utilized in a variety of contexts and situations beyond the standard therapy practice. Educators and schools in particular are looking to include yoga as a cost-effective, evidence-based component of urgently needed wellness programs for their students. *Objectives*. The primary goal of this study was to systematically examine the available literature for yoga interventions exclusively in school settings. The objective of this report was to explore the evidence of yoga-based interventions on academic, cognitive, and psychosocial benefits. *Methods*. Studies were identified by searching PubMed, PsycInfo, Embase, ISI, and the Cochrane Library. An extensive search was conducted for studies published between 1980 and October 31, 2014, using the following terms or key words, *yoga*, *school*, *education*, and *children*, alone and in combination with additional terms such as *program*, *intervention*, and *yoga-based*. *Results and Conclusions*. Forty-eight studies were identified and nine randomized control trials met criteria for inclusion in this review. Although most of the studies were classified as 2b, according to AHRQ evidence level criteria, which means low quality randomized control trials, this review suggests beneficial effects of yoga-based interventions at school on both psychological and cognitive functions. Effect size was found for mood indicators, tension and anxiety in the POMS scale, self-esteem, and memory. Future research requires greater standardization and must deal with the problem of appropriateness: what type of yoga-based intervention is most suitable for children and at what frequency and duration and observed variables.	1

*Introduction *			
Rationale	3	Yoga is a holistic system of varied mind-body practices that can be used to improve mental and physical health. Due to the well-known restorative effects of yoga on mental health, it has been utilized in a variety of contexts and situations beyond the standard therapy practice. Educators and schools in particular are looking to include yoga as a cost-effective, evidence-based component of urgently needed wellness programs for their students. However, there is no critically appraised evidence such as systematic reviews on potential benefits of yoga-based interventions in school settings.	2
Objectives	4	The objective of this report was to review methodological quality among selected studies, exploring the evidence of yoga-based interventions regarding academic, cognitive, and psychosocial benefits, and to contribute to the study of low-cost, health-focused alternative programs for children and adolescents in school settings.	2

*Methods *			
Eligibility criteria	5	Peer-reviewed, published manuscripts were considered. Studies were selected if (1) they included a yoga or yoga-based intervention, (2) the intervention was restricted to school settings (integrated into the school schedule or after class), (3) they included children and adolescents (ages 5–18), (4) they included an evaluation of anxiety, depression, stress, or other psychological measures such as mood indicators, self-esteem, confidence, and quality of life at both preintervention and postintervention, (5) they included the assessment of academic or cognitive performance as a consequence of the yoga intervention (pre- and postintervention), (6) the research designs were pilot studies, quasi-experimental designs, or randomized designs and included control groups with no interventions or an active control (comparative intervention), and (7) they were written in English. Exclusion criteria comprised (1) studies that utilized only meditation or relaxation techniques without the physical components such as postures (*asana*) or controlled breathing (*pranayama*) (MBSR based programs usually have yoga as part of the practice, but only MBSR studies in which yoga is highlighted or is the main component of the program were included in this review, whose intention is to cover primarily yoga), (2) yoga-based programs for children with learning disabilities or any diagnosed mental disorder, and (3) dissertations and conference presentations.	5
Information sources	6	Literature searches were conducted in PubMed, PsycInfo, Embase, ISI, and the Cochrane Library (1980–2014). An extensive search was conducted for studies published between 1980 and October 31, 2014, using the following terms or key words, *yoga*, *school*, *education*, and *children*, alone and in combination with additional terms such as *program*, *intervention*, and *yoga-based*. Ongoing registered clinical trials were not searched. The details for the full search strategy were listed in a flow diagram, as shown in Figure 1.	5
Search	7	PubMed, EMBASE, and the Cochrane Central Register of Controlled Trials (CENTRAL) in the Cochrane Library	5
Study selection	8	Four authors conducted the literature searching (C. Ferreira-Vorkapic, M. Marchioro, and S. Telles) and study selection (C. Ferreira-Vorkapic, M. Marchioro, and E. Kozasa).	5
Data collection process	9	Authors (C. Ferreira-Vorkapic, M. Marchioro, S. Telles, and E. Kozasa) screened abstracts to identify articles that meet inclusion criteria. Potential articles were then evaluated for inclusion. To conduct the study, all data was collected and evaluated in terms of selection criteria, procedure, participants, intervention, methodology, assessment tools, and outcomes. Follow-up and results were also assessed. The review has been prepared using preferred reporting criteria for systematic review guidelines (PRISMA). J. M. Feitoza has performed all the statistics (effect size). For the effect size calculation, the means and standard deviations (postintervention) from experimental and control groups were obtained directly from the studies.	5
Data items	10	Yoga, school, education, and children.	5
Risk of bias in individual studies	11	After the first appraisal, another examiner (J. Simões) evaluated the selected RCTs and a *kappa* coefficient was calculated in order to estimate reliability of data collection methods. The observed kappa score of *r* = 0.9 suggests great consistency of agreement between investigators.	6
Effect size calculation	12	The comparison between experimental conditions was carried out after the analysis of the pooled effect size by the generic inverse variance method (random effect model) through standardized mean difference and Hedges' g. Ninety-five percent confidence intervals were computed for all variables. Two studies [19, 26] were excluded from the effect size analysis due to insufficient data (mean values not provided). The analysis of the effect size of specific measures could only be performed on studies that observed the same variables such as mood, tension and anxiety [20, 23], self-esteem [23, 27], and memory [28, 29] when comparing the yoga to control groups.	6
Summary measures	13	The quality and reliability of the randomized control trials (RCTs) were evaluated according to the evidence levels recommended by the Oxford Center for Evidence-Based Medicine (AHRQ, 2002). The items included study question, study population, randomization, blinding, interventions, outcomes, statistical analysis, results, discussion, and funding source. The quality of all the included trials was categorized into levels of evidence varying from 1 to 5, according to low, unclear, or high risk of bias. The quality and reliability of the randomized control trials (RCTs) were evaluated according to the evidence levels recommended by the Oxford Center for Evidence-Based Medicine. Table 1 shows the classification of scientific evidence in systematic literature reviews	6

*Results *			
Study selection	14	A flow chart depicted the search process and study selection (Figure 1). Of the 48 studies identified, 9 randomized control trials met criteria for inclusion in this review. Generally, the RCTs had low AHRQ evidence scores, with most studies receiving a score of 2b due to the lack of blindness and follow-up, reflecting the regular quality of reporting in these studies. All results and conditions of the studies are summarized in Table 3.	6
Study characteristics	15	The methodological quality of most included trials was generally reduced. The details are as shown in Table 3. The randomized allocation of participants was mentioned in all trials. Blinding information, however, was insufficient due to the nature of the study (in yoga trials practitioner blinding is impossible). Interventions included yoga or yoga-based programs conducted exclusively in school settings for children ranging from 7 to 17 years. Comparison groups included physical education, Ayurvedic treatment, and control. Cognitive and psychological functions were assessed and the total treatment duration ranged from 8 to 18 weeks.	6
Risk of bias within studies	16	The number of trials was too small to conduct any sufficient additional analysis of publication bias.	6
Effect size results	17	After an overall effect size calculation of each study (except for Ramadoss and Bose, 2010 [19], and White, 2012 [26]), the effect sizes obtained from similar measures were grouped together: mood, tension, anxiety, self-esteem, and memory. Figure 2 shows the forest plot of the general effect size in the selected studies. The general plot shows divided results with half of the studies favoring yoga and the other half favoring control. *P* value for overall effect is not significant (*p* = 0.91), but this is probably due to heterogeneity of the variables. Effect size from mood state indicators (POMS) was calculated from Khalsa et al., 2012 [23], and Noggle et al., 2012 [20]. Results indicate that the yoga group showed significantly better scores in the postintervention condition (*p* = 0.02) (Figure 3). The same scale shows a second significant effect for the subitems tension and anxiety also after the yoga practice (*p* = 0.04) (Figure 4). A third comparison was carried out for the variable self-esteem [23, 27]. Results show greater self-esteem perception in the postintervention condition for the yoga group (*p* = 0.04) (Figure 5). Effect size for memory was also analyzed in Sarokte and Rao 2013, [28] and Verma et al., 2014 [29]. Both studies utilized memory assessment tools, but Sarokte and Rao 2013, [28] used two different instruments. The results show increased memory performance for the yoga group (*p* < 0.00001) (Figure 6).	7
Synthesis of results	18	Regarding the effects of yoga on psychological well-being, of the six studies, three of them support the benefits of yoga or yoga-based programs for children in school settings. Khalsa et al. [23] observed that yoga participants showed statistically significant differences over time relative to controls on measures of anger control and fatigue/inertia. Noggle et al. [20] also observed preventive benefits in psychosocial well-being (anxiety and negative affect) for students enrolled in a yoga program. In Ramadoss and Bose [19], only the yoga group demonstrated a slight decrease in stress while maintaining self-control. In contrast, Haden et al., 2014 [30] and White [26] observed a significant increase in perceived stress in the yoga group compared to the physical education and control groups, respectively. However, in White [26], both groups (yoga and control) reported significantly greater self-esteem and self-regulation over time. Lastly, Hagins et al. [32] found that yoga did not reduce stress reactivity compared to a physical education class when students were submitted to stressor tasks. Effect size was found for mood indicators, anxiety and tension (POMS), self-esteem, and memory. The RCT of each article is described in detail in the text.	7

*Discussion *			
Summary of evidence	19	Although most of the studies were classified as 2b, according to AHRQ evidence level criteria, which means low quality randomized control trials, this review shows beneficial effects of yoga-based interventions at school on both psychological and cognitive functions (effect size was found for mood indicators, anxiety and tension, self-esteem and memory), but the negative effects of yoga were also observed in some of the studies and might be explained to some extent by the adaptation process by children, the absence of attentional control, and the inadequacy of yoga practice for children. Future research requires greater standardization and must deal with the problem of appropriateness: what type of yoga-based intervention is most suitable for children and at what frequency and duration and observed variables.	16
Limitations	20	Not only is the number of RCTs low, but also the trials were of reduced methodology quality and had risk of bias in terms of design, reporting, and methodology. It is comprehensible that it is difficult to perform double-blinding studies with yoga, but blinding to the outcome assessors and data analyzer could be feasible and has not been reported. One limitation of this review is that it was not possible to calculate the effect size of all variables observed in the selected studies due to their heterogeneity.	16
Conclusions	21	This review analyzed nine peer-reviewed RCT studies, in which yoga was taught to children in a school setting. Outcome measures included psychological well-being and cognitive functions, such as attention and memory. Effect size was found for mood indicators, anxiety and tension (POMS), self-esteem, and memory. While supportive in many studies, the utility of yoga in educational settings is inconclusive due to the small number of randomized control trials in the literature. Even though only RCTs were reviewed, methodological and statistical problems might have contributed to the uncertainty: inadequate sample sizes, absence of control groups, variability in the type of yoga being taught, long duration of yoga sessions, inappropriate psychometric tools for children, and failure to measure intervening variables such as mindfulness and body awareness, which are important parts of yoga practice. This review suggests valuable effects of yoga-based interventions at school on both psychological status and cognitive function in some studies but future research requires greater standardization and must deal with the problem of appropriateness; what type of yoga-based intervention is most suitable for children, specifically in terms of the frequency and duration?	22

*Funding *			
Funding	22	This research was funded by the FAPITEC Agency under Process no. 7838.UNI321.21944.25062013.	22

Adapted from [24].

**Table 3:** Selected randomized control trials (RCTs).

Study	Sample	Program	Intervention	Variables	Evaluation tools	Results	Evidence level
Hagins et al., 2013^*∗*^ [32]	Middle school students, ages 11 to 12 (*n* = 30)	Vinyasa Yoga 15 weeks 50 minutes, three times a week	Yoga or physical education (c)	Blood pressure (BP), heart rate (HR), and behavioral stressor tasks (mental arithmetic and Mirror Tracing Tasks).	Automated blood pressure cuff, Mental Arithmetic Task (MAT), and Mirror Tracing Task (MTT).	There were no significant differences between groups.	2b (not double blinded, no follow-up)

Haden et al., 2014^*∗*^ [30]	Middle school students, ages 11 to 12 (*n* = 30)	Ashtanga Yoga 12 weeks 90 minutes, three times a week	Yoga or physical education (c)	Emotional (affect and self-perceptions) and behavioral variables (internalizing and externalizing problems and aggression).	PANAS, Child Behavior Checklist (CBCL), Revised Parent Rating Scale for Reactive and Proactive Aggression (R-PRA), and the Self-Perception Profile for Children (SPPC).	There were no significant changes between groups in self-reported positive affect, global self-worth, aggression indices, or parent reports of their children's externalizing and internalizing problems. However, negative affect increased for those children participating in yoga when compared to the PE program.	2b (insufficient blinding, no follow-up)

Khalsa et al., 2012 [23]	High school students, ages 12 to 13 (*n* = 136)	Yoga Ed 11 weeks 30 to 40 minutes, two to three times a week	Yoga or physical education (c)	Mood, anxiety, perceived stress, resilience, and other mental health variables.	The Self-Report of Personality (SRP) version of the Behavior Assessment Survey for Children Version 2 (BASC-2), POMS, the Resilience Scale (RS), and the Perceived Stress Scale (PSS).	Yoga participants showed statistically significant differences over time relative to controls on measures of anger control and fatigue/inertia. Most outcome measures exhibited a pattern of worsening in the control group over time, whereas changes in the yoga group over time were either minimal or showed slight improvements.	2b (not double blinded, no follow-up)

Noggle et al., 2012 [20]	High school students, ages 16 to 17 (*n* = 51)	Kripalu Yoga 10 weeks 30 minutes, two to three times a week	Yoga or physical education (c)	Psychological well-being, mood and negative affect (primary measures), stress, resilience, anger, and acceptance (secondary measures).	Primary outcomes: Profile of Mood States-Short Form and Positive and Negative Affect Schedule for Children. Secondary outcomes: Stress Scale and Inventory of Positive Psychological Attitudes, Resilience Scale, State Trait Anger Expression Inventory-2, and Child Acceptance Mindfulness Measure.	Mood improved in yoga and worsened in controls. Negative affect worsened in controls and improved in yoga.	2b (not double blinded, no follow-up)

Ramadoss and Bose, 2010 [19]	High school students, ages or grades not informed (*n* = 472)	Niroga TLS 18 weeks 15 minutes, from one to five times a week	Yoga once a week, yoga twice a week, yoga three times a week, yoga five times a week, or a waiting list group (c).	Stress and self-control.	Perceived Stress Scale (PSS-10) and Tangney's Self-Control Scale (TSCS-13).	The intervention group demonstrated a slight decrease in stress and maintenance in self-control. In contrast, the control group, which received no classes, demonstrated no significant change in stress and a nonsignificant trend toward deterioration of self-control.	2b (not double blinded, no follow-up)

White, 2012^*∗*^ [26]	Elementary school students, ages 9 to 11 (girls) (*n* = 155)	The Mindful Awareness for Girls through Yoga 8 weeks 60 minutes, once a week (10-minute homework for the other 6 days of the week)	Yoga or no intervention (c).	Stress, coping strategies, self-esteem, and mindfulness.	The Feel Bad Scale, the Schoolagers' Coping Strategies Inventory, and the Healthy Self-Regulation Subscale of the Mindful Thinking and Action Scale for Adolescents.	No significant differences between groups were found. Over time, the intervention group was more likely than the control group to report stress	2b (not double blinded, no follow-up)

Sarokte and Rao, 2013 [28]	Middle and high school students, ages 10 to 16 (*n* = 90)	Hatha Yoga 3 months 40 minutes, every day as homework	Medhya Rasayana (Ayurveda treatment), Yoga, or no intervention (c)	Executive functions and mental status.	Short-term memory test, pictures, serial effects test, words, and mini mental state scale.	Group B showed highly significant and most effective changes in short-term memory test pictures and serial recall effects test using memory scope. Group C showed highly significant and most effective changes with respect to subjective and objective parameters in mini mental status scale.	2b (not double blinded, no follow-up)

Telles et al., 2013 [27]	Elementary and middle school students, ages 8 to 13 (*n* = 98)	Hatha Yoga 3 months 45 minutes, five times a week	Yoga or physical activity (c)	Self-esteem, attention, and physical fitness.	Stroop color-word task for children, Battle's self-esteem inventory, and the teachers' rating of the children's obedience, academic performance, attention, punctuality, and behavior with friends and teachers.	Teachers' rating of the children's behavior. Social self-esteem: ↑ PA group Stroop: ↑ both groups (higher interference in the PA group). Total, general, and parental self-esteem improved in the yoga group. Both groups showed an increase in BMI and number of sit-ups. Balance worsened in the physical exercise group, while plate tapping improved in the yoga group.	2b (not double blinded, no follow-up)

Verma et al., 2014 [29]	Middle and high school students, ages 11 to 15 (*n* = 82)	Hatha Yoga 12 weeks 45 minutes, five times a week	Yoga and control groups	Cognitive functions.	Mental Ability Test and Battery and Memory Test.	Significant improvement was observed in measures of mental ability and memory in experimental group	2b (not double blinded, no follow-up)

^*∗*^Negative effects on stress and affect or no significance between groups.
